# Assessing Budding Yeast Phosphoproteome Dynamics in a Time-Resolved Manner using TMT10plex Mass Tag Labeling

**DOI:** 10.1016/j.xpro.2020.100022

**Published:** 2020-06-03

**Authors:** Andrew W. Jones, Helen R. Flynn, Frank Uhlmann, Ambrosius P. Snijders, Sandra A. Touati

**Affiliations:** 1 Mass Spectrometry Proteomics Science Technology Platform, The Francis Crick Institute, London NW1 1AT, UK; 2 Cell Cycle Laboratory, The Francis Crick Institute, London NW1 1AT, UK; 3 Chromosome Segregation Laboratory, The Francis Crick Institute, London NW1 1AT, UK; 4 Institut de Biologie Paris Seine, CNRS UMR7622, Sorbonne Université, Paris, France

## Abstract

Amine-reactive Tandem Mass Tag 10plex (TMT10plex) labeling permits multiplexed protein identification and quantitative analysis by tandem mass spectrometry (MS/MS). We have used this technology to label 20 *Saccharomyces cerevisiae* samples collected in a time-resolved manner from a wild-type and phosphatase mutant background to characterize phosphoproteome dynamics. Here, we provide a detailed protocol for biological and mass spectrometry sample preparation and analysis. For complete details on the use and execution of this protocol, please refer to Touati et al. (2019).

## Highlights

• Characterization of the phosphoproteome in a time-resolved manner
• TMT10plex Mass Tag labelling of budding yeast extracts
• Mass spectrometry sample preparation and analysis

## Before You Begin

### Cell Culture and Synchronization

**TIMING: 2 days**

**Figure 1:**
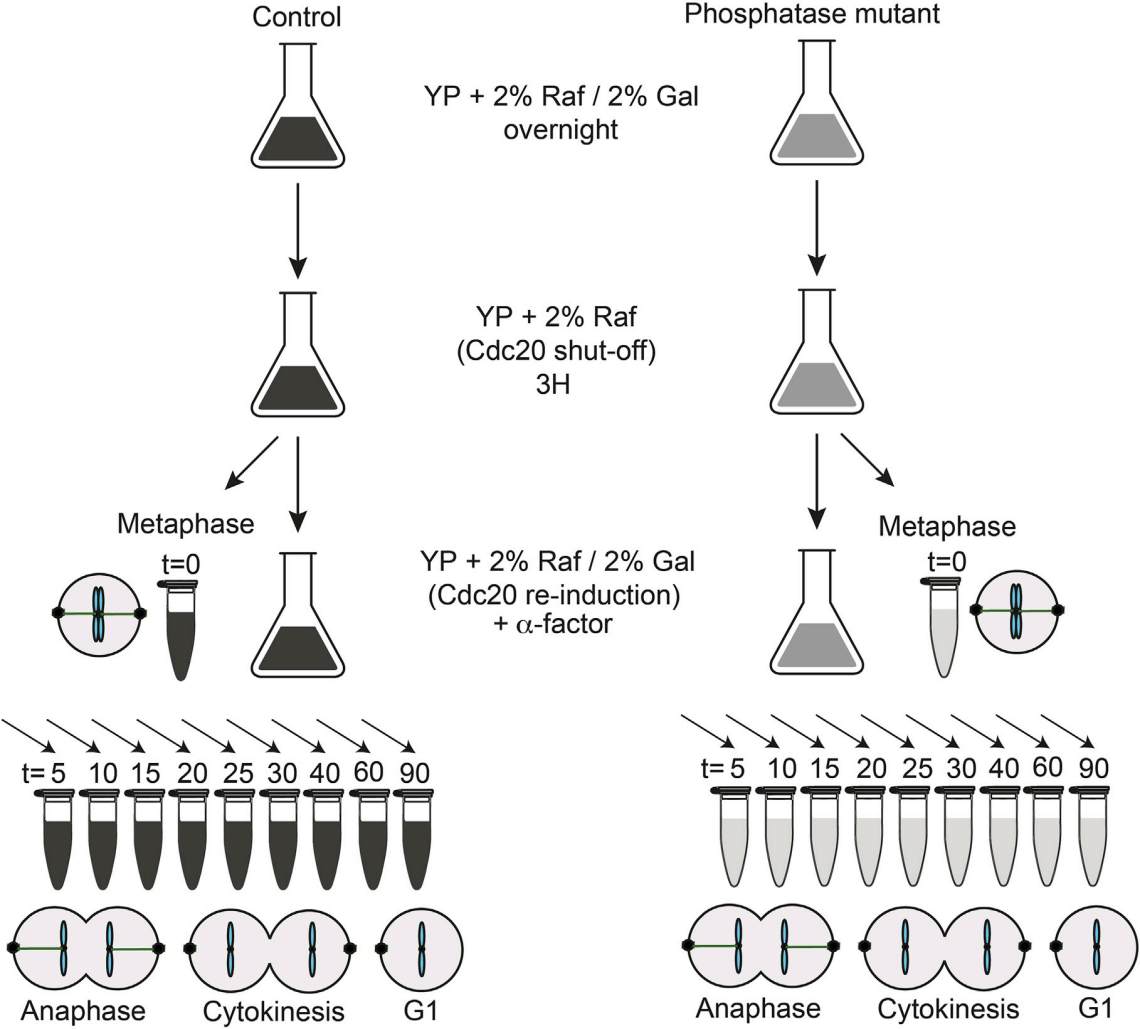
Cell Synchronization and Collection

***Note:*** A stock solution of 20% galactose and 20% raffinose is used.

1. Grow control and mutant cells for 16 hours in 400 ml of YP media (Rose et al., 1990) containing 2 % galactose and 2 % raffinose at 25°C.
2. In the morning, back dilute the cells to OD_600_=0.1 (around 1.5∗10^6^ cells/ml) and grow them in YP medium containing 2 % galactose and 2 % raffinose at 25°C. At OD_600_=0.2 (around 3∗10^6^ cells/ml), filter the cells, wash with 3 l of YP galactose-free medium and grow them for 3 h in YP medium containing 2 % raffinose at 25°C. This results in a metaphase cell cycle arrest due to Cdc20 depletion.
3. Cells should have reached OD_600_=0.3-0.4 (around 4.5-6∗10^6^ cells/ml). Collect 25 ml for the first time point (t=0) just before galactose re-addition and process the sample immediately (see 2. Extract preparation).
4. Add 2 % of galactose to both control and mutant cells to re-induce Cdc20 expression and synchronous progression through the mitotic stages before returning to G1. Add also 0.8 μg/ml of α-factor to re-arrest cells in G1 at the end of the experiment.
5. Collect time-point samples at t=5, 10, 15, 20, 25, 30, 40, 60 and 90 min in control and mutant strains and process the samples immediately (Figure 1).

**CRITICAL:** A high level of cell cycle synchrony is essential when performing global phosphoproteome analysis in a time-resolved manner. We used *Saccharomyces cerevisiae* cells expressing the anaphase promoting complex/cyclosome (APC/C) co-activator Cdc20 under control of the galactose-inducible *GAL1* promoter. Both control and phosphatase mutant cells harbor this modified version of the *CDC20* gene, allowing cell cycle arrest in metaphase and synchronous release into progression through anaphase and mitotic exit (Uhlmann et al., 1999).

***Alternatives:*** To synchronize the cells in metaphase, the gene Cdc20 can be placed under control of the methionine-repressible MET3 promoter. In this case, cells have to be grown for 16 hours in minimal media without methionine.

### Extract Preparation

**TIMING: 3 h**

6. Centrifuge samples at 2000 g for 3 min and discard the supernatant.
7. Re-suspended the pellet in 10 ml of ice-cold 20 %TCA, vortex and keep on ice for at least 1 h. The samples from throughout the timecourse analysis will be collected at this stage. Then all samples are further processed together.
8. Remove the TCA by aspiration and wash the pellets twice with 10 ml of cold acetone (kept at -20 ˚C). Following the final acetone wash, ensure that any remaining acetone has evaporated before continuing to the next step.
9. Resuspend the samples in 400 μl of lysis buffer (8 M urea, 50 mM HEPES pH 8.2, 5 mM EDTA, 1 mM dithiothreitol, 50 mM sodium fluoride, 1 mM sodium vanadate, 1 mM PMSF, cOmplete EDTA-Free Protease Inhibitor Cocktail) and transfer the samples into 2 ml Eppendorf tubes suitable for use in a cell breaker.
10. Add 500 μl acid-washed glass beads and beat on a Fast-Prep cell breaker in the cold room. Program: 6.0 m/s - 4 x 40 s. Ensure that lids remain safely closed between the breakage rounds. Confirm efficient cell breakage using a phase contrast microscope where intact cells appear bright while broken cells appear dark.
11. Punch a hole with a heated hypodermic needle to the bottom of the cell breaker tubes, place into a 15 ml centrifuge tube and collect the extracts by brief centrifugation.
12. Transfer the extracts into 1.5 ml test tubes and centrifuge at 16000 g for 10 min.
13. Retrieve the supernatants and measure the protein concentration using Protein Assay Dye. The protein concentration should be >500 μg/ml.
14. Transfer a volume corresponding to 200 μg of protein into LoBind microcentrifuge tubes and freeze at -80 °C.

**PAUSE POINT:** Samples can be stored at -80 °C indefinitely.

***Alternatives:*** The liquid culture can be precipitated directly in TCA 100% with a ratio 1 per 5. Simplified lysis buffer containing 8 M urea, 5 mM EDTA pH 7.5 and 50 mM ammonium bicarbonate has also been used.

***Note:*** Primary amines interfere with the TMT labeling, so the lysis buffer has to be free of reactive amines (*e.g.* Tris), hence it is recommended to use buffers such as HEPES or triethylammonium bicarbonate.

***Note:*** Detergents can interfere with LC-MS/MS-based analysis, so the lysis buffer should be free of detergents (*e.g.* sodium dodecyl sulfate). If this is not possible, then alternative sample cleaning methods, *e.g.* SP3 (Hughes et al., 2019) or FASP (Wiśniewski et al., 2009), may be employed.

***Note:*** The remainder of the material can be retained to perform additional experiments, e.g. western blotting, as applicable.

### Key Resources Table

**Table:** 

REAGENT or RESOURCE	SOURCE	IDENTIFIER
**Chemicals, Peptides, and Recombinant Proteins**		

α-factor	Peptide Chemistry Science Technology Platform, The Francis Crick institute Sequence: NH_2_-WHWLQLKPGQPMY-COOH	N/A
cOmplete EDTA-Free Protease Inhibitor Cocktail	Sigma-Aldrich	Cat# 04693132001
Protein Assay Dye	Bio-Rad	Cat# 5000006
Trichloroacetic acid	Sigma-Aldrich	Cat# T0699
Urea	Fisher Scientific	Cat# BP169
HEPES pH 8.2	Sigma-Aldrich	Cat# H3375
EDTA	Sigma-Aldrich	Cat# E9884
Sodium fluoride	Sigma-Aldrich	Cat# S1504
Sodium vanadate	New England BioLabs	Cat# P0758
PMSF	Sigma-Aldrich	Cat# P7626
Pierce Trypsin Protease	ThermoFisher	Cat# 90058
Dithiothreitol	Sigma-Aldrich	Cat# D5545
Iodoacetamide	Sigma-Aldrich	Cat# A3221
HEPES pH 8.5	Fisher Scientific	Cat# 15433969
Trifluoroacetic acid	Fisher Scientific	Cat# A116
Acetonitrile	Fisher Scientific	Cat# A955
Acetic acid	Fisher Scientific	Cat# A11350
50 % Hydroxylamine	ThermoFisher	Cat# 90115
Formic acid	Fisher Scientific	Cat# A117
DMSO	Honeywell	Cat# 41640
Water	Fisher Scientific	Cat# W6

**Critical Commercial Assays**		

TMT10plex Isobaric Label Reagent Set 1 x 0.8 mg	ThermoFisher	Cat# 90110
Pierce TiO2 Phosphopeptide Enrichment Spin Kits	ThermoFisher	Cat# 88303
High-Select Fe-NTA Phosphopeptide Enrichment Kit	ThermoFisher	Cat# A32992

**Experimental Models: Organisms/Strains**		

*Saccharomyces cerevisiae* strains W303	https://doi.org/10.17632/tspgywx7g3.1	N/A

**Software and Algorithms**		

Perseus v1.4.0.2	Perseus	https://maxquant.net/perseus/
MaxQuant v1.5.0.13	MaxQuant	https://www.maxquant.org

**Other**		

Fast-Prep cell breaker	N/A	N/A
Refrigerated centrifuge capable of achieving 18,000 ×*g*	N/A	N/A
Benchtop centrifuge	N/A	N/A
Spectrophotometer	Ultrospec 7000	Cat# 29003605
Heat block (37°C and 56°C)	N/A	N/A
Thermomixer	N/A	N/A
SpeedVac vacuum concentrator	N/A	N/A
Ultrasonic water bath	Ultrawave	Cat# U300
Sep-Pak C_18_ Vac Cartridges	Waters	Cat# WAT054955
Vacuum manifold	Phenomenex	Cat# AH0-6024
Empore C_18_ membrane	Supelco	Cat# 66883-U
UltiMate 3000 HPLC System	Thermo Scientific	N/A
EASY-Spray C_18_ column 75 μm × 50 cm	ThermoFisher	Cat# ES803
Orbitrap Fusion Lumos Tribrid Mass Spectrometer	Thermo Scientific	N/A
Total recovery glass autosampler vials	Waters	Cat# 186005663CV

## Step-by-Step Method Details

### Protein Digestion

**TIMING: 18–24 h**

As this protocol is interested in identifying and quantifying individual phosphosites, a bottom-up approach is being undertaken. The initial step of the vast majority of bottom-up proteomic approaches requires the digestion of proteins into peptides, in this case, we are using the widely used enzyme trypsin which cleaves C-terminal to lysine and arginine residues.

1. Thaw the samples and treat each lysate with 1 μl of 1 M dithiothreitol for 25 min at 56 °C to reduce disulfide bonds between cysteine residues. Cool samples to ∼22 °C before proceeding to the next step.
2. Alkylate the cysteine sulfhydryls using 4 μl of 500 mM iodoacetamide for 30 min at ∼22 °C, protected from light. Iodoacetamide has to be in excess compared to the dithiothreitol. This step prevents the reformation of disulfide bonds between cysteine residues.
3. Quench the reaction with 1.5 μl 1 M dithiothreitol to stop the alkylation reaction.
4. Before preforming the proteolytic digestion, the concentration of urea must be reduced to <2 M. Add four sample volumes of 50 mM HEPES pH 8.5. (e.g. 200 μl sample:800 μl 50 mM HEPES pH 8.5).
5. Add sequencing grade trypsin at 2 % of total protein (4 μg/sample) and incubate the reaction at 37 °C for 6-18 h. Trypsin cleaves peptides on the C-terminal side of lysine and arginine amino acid residues (except when proline is the next residue).

***Note:*** Trypsin digestion results in peptides with an average of 14 amino acids in length, which is favorable for LC-MS/MS analysis. Other enzymes, *e.g.* lysyl endopeptidase (LysC), may be used in place of, or in parallel to, trypsin. LysC cleaves C-terminal to lysine residues which ensures all peptides contain two primary amine groups which are the acceptors for TMT labelling.

### C_18_ Spin Column Cleanup of Proteolytic Digests

**TIMING: 3–4 h**

Before performing TMT labeling, components of the lysis and digestion buffer need to be removed using solid phase extraction. To concentrate and clean up the samples a C_18_ SepPak 130 mg bed volume is used for each sample in conjunction with a vacuum manifold set at -2 psi, to slowly pass each solution below through the solid phase extraction device.

***Alternatives:*** C_18_ MacroSpin column (Nest Group) in conjunction with a centrifuge can be used to replace the C_18_ SepPak column and vacuum.

6. Centrifuge the samples briefly to collect any condensate liquids on the lid.
7. To prepare the samples for desalting, acidify each digested sample with trifluoroacetic acid (TFA) to a concentration of 0.4 % (v/v) and ensure that the pH is ≤2.
8. Wash the columns with 1.25 ml of acetonitrile and condition them with 450 μl of 50 % acetonitrile / 0.5 % acetic acid. For all the steps, make sure that the entire solution passes through the device, but without drying the column.
9. Equilibrate the columns with 1.25 ml of 0.1 % TFA. Discard the flow through.
10. Load the samples onto the columns. Save flow-through.
11. Wash the columns with 125 μl of 0.5% acetic acid. Combine the flow through with the flow through from Step 5, retain the SepPak and store at -20 °C, in case of loss of sample due to unforeseen technical or user error.
12. Elute the peptides from the columns by adding 750 μl of 50 % acetonitrile with 0.5 % acetic acid.
13. Dry the eluted peptides by vacuum centrifugation (∼2 h, ambient). Store the dried samples at -80 °C.

**PAUSE POINT:** Dried peptides can be stored at -80 °C indefinitely.

***Note:*** It is recommended to store the flow through fractions until peptide recovery has been confirmed in subsequent steps.

### TMT10plex Mass Tag Labelling

**TIMING: 2 h**

**Figure 2:**
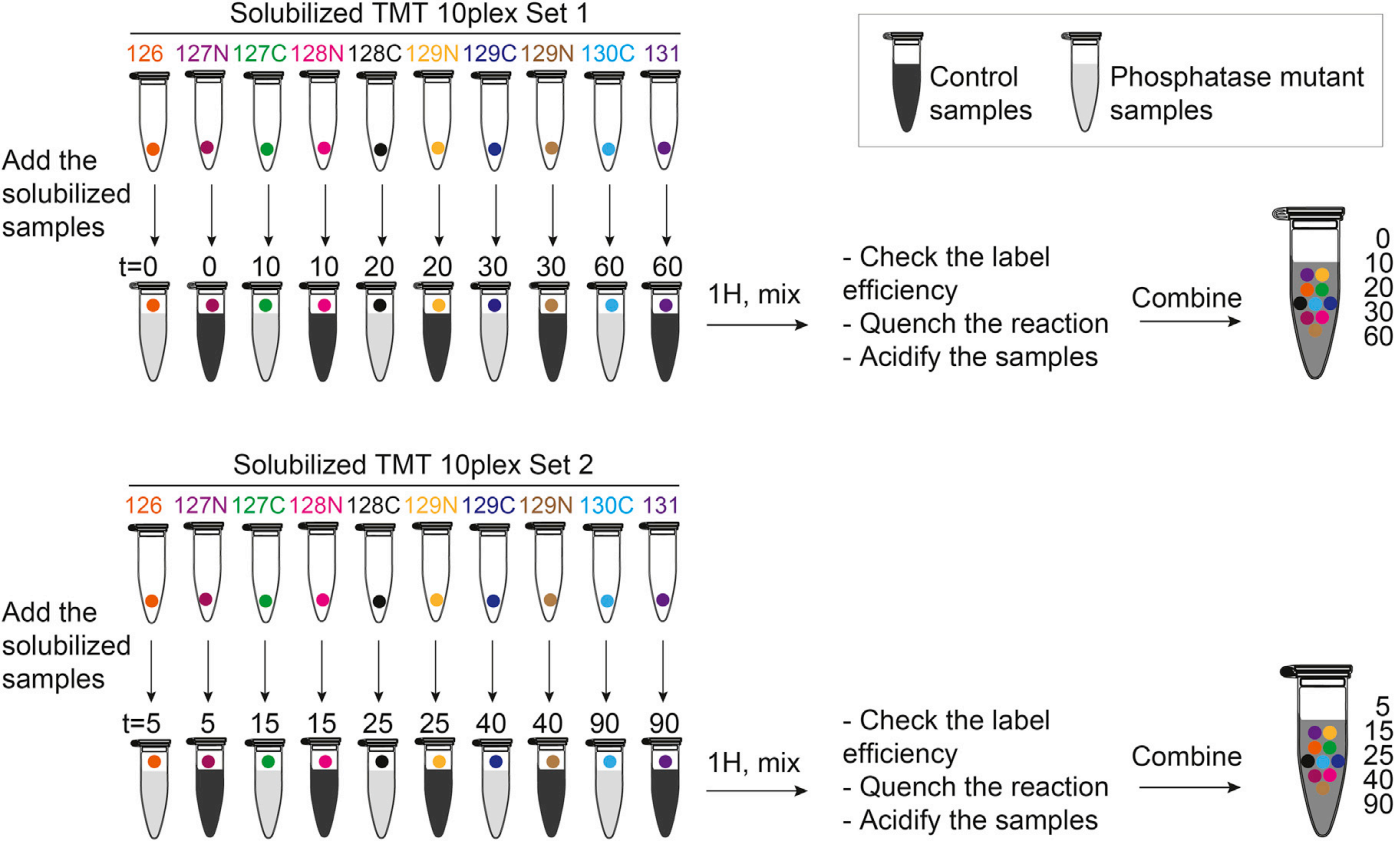
Labeling with TMT10plex Isobaric Mass Tag Reagents Sets

The TMT10plex reagent set contains ten different isobaric compounds with the same nominal mass and chemical structure. A mass reporter is linked via a spacer arm to an amine-reactive NHS-ester group. By using tandem mass spectrometry, TMT10plex allows the identification and relative quantification of ten different samples simultaneously. Two sets of TMT10plex (0.8 mg per tag) are required to label the 20 samples (i.e. 10 controls and 10 phosphatase mutants) as shown in Figure 2. The protocol follows the steps of the manufacturer but is adapted to label 200 μg of peptide material per channel.

***Alternatives:*** The TMT kit is also available in 6-, 11- or 16- plexing capacity. Alternative isobaric labeling reagents include iTRAQ.

14. Solubilize each dried sample in 200 μl of 50 mM HEPES pH 8.5 by sonication in an ultrasonic bath for 10 min. Check that the pH is ∼8.5 by spotting 0.2 μl on a pH indicator strip. If necessary adjust the pH by adding 1 M HEPES pH 8.5, and add equivalent volumes of 50 mM HEPES to the other samples to ensure that the peptide concentration is equal.
15. Equilibrate the TMT10plex Isobaric Label Reagent at ∼22°C for 15 min and briefly centrifuge the reagent tubes.
16. Solubilize each vial of isobaric labels in 40 μl anhydrous acetonitrile carefully and keep the tubes closed as much as possible. Briefly centrifuge and use the TMT reagents immediately.
17. Add the sample solution to the reagent tubes according to the scheme in Figure 2. Vortex and briefly centrifuge before incubating for 1 h at ∼22 °C on a Thermomixer set at 750 rpm. If Thermomixer is not available, vortex the samples occasionally during the 1 h incorporation
18. Centrifuge the tubes briefly and retrieve 2 μl of each of the 20 samples and dilute in 18 μl of 0.1% TFA (i.e. 20 individual diluted samples). Use these samples to determine the labeling efficiency as described in section 4.
19. Make two groups of 10 samples following the scheme in Figure 2. Then, combine 5 μl each of the 10 samples in both sets, resulting in two mixed samples. Vortex and centrifuge briefly. Dilute 2 μl of each the two mixes in 18 μl of 0.1% TFA and use these samples in the mixing check as described in section 4. Store the remainder of each labelled sample immediately at -80 °C.

***Note:*** To allow a relative comparison of the phosphoproteome in the wild type and mutant context, it is essential that one half of the TMT10plex set labels the control samples and the other half labels the phosphatase mutant samples.

***Note:*** Every TMT lot has a different correction error, which can be found in the "reporter ion isotopic distribution" section of the product data sheet. It is important to keep a note of the correction error for the later data analysis.

**CRITICAL:** Do not quench the reaction until labeling has been confirmed by LC-MS/MS.

### Evaluation of TMT10plex Isobaric Mass Tag Labeling Efficiency and Sample Mixing by LC-MS/MS

**TIMING: 12 h**

The following steps are illustrative of our specific LC-MS/MS protocol and instrument; however, any mass spectrometer with beam-type CID and MS2 resolution ≥35,000 coupled to liquid chromatography and any database search algorithm capable of processing TMT labeled data should be suitable.

20. Transfer the 20 individual diluted samples and the two mixed samples to total recovery glass autosampler vials to check the enrichment efficiency. The liquid needs to be in the bottom of the vial and must not contain any air bubbles.

Inject 10 μl of each diluted TMT labelled sample or mixture on an UltiMate 3000 HPLC system coupled to an Orbitrap Fusion™ Lumos™ Tribrid™ Mass Spectrometer. Set the nanoLC flow rate to 250 nl/min. Separate the peptides using the following gradient: 0-5 min (2 % mobile phase B), 33 min (40 % mobile phase B), 34-44 min (95 % mobile phase B), 45-60 min (2 % mobile phase B). (Phase A: 95 % H_2_O, 5 % DMSO, 0.1 % Formic Acid, Phase B: 75 % Acetonitrile, 5 % DMSO, 20 % H_2_O, 0.1 % Formic Acid).

21. Use the following instrument method for the mixtures. MS settings: Orbitrap mass analyser (120000 resolution), scan range 400-1400 *m*/*z*, AGC target 4.0×10^5^, maximum injection time 100 ms. Data dependent mode cycle time of 3 s between MS scans. MS2 settings: Orbitrap mass analyser 60000), activation type HCD, HCD collision energy 38 %, isolation window 0.7 *m*/*z*, first mass 100 *m*/*z*, AGC target 1.0×10^5^, maximum injection time 105 ms, include charge states 2-6, dynamic exclusion 30 s after 1 time.
22. A more detailed discussion regarding data acquisition strategies for phospho-TMT experiments is provided by Jiang et al, 2017.
23. Perform a MaxQuant database search using an up to date *S. cerevisiae* proteome FASTA database (freely available from the UniProt website; http://www.uniprot.org/proteomes/) for the 20 label check samples (http://www.maxquant.org and guidelines to search process). Ensure that TMT10plex labels (+229.1629 Da on peptide N-termini and Lysine side chains) are selected as variable modifications.
24. To ensure that peptides have been optimally labelled, fewer than 5 % of the identified peptides within the modificationSpecificPeptides.txt output should not have a TMT10plex-Nter and/or TMT10plex-Lys variable modification. If this criterion is not reached for a specific sample, then a second round of labelling is recommended.
25. Perform a MaxQuant database search using an up to date *S. cerevisiae* proteome database for the two mixing checks. Ensure that Reporter ion MS2 with TMT10plex for quantification is selected. During the MaxQuant analysis, the correction numbers of the TMT lot need to be taken into consideration. At this stage, difference in the quantity of proteins between each sample can be observed and will be adjusted during the mixing of the original samples (see point 6).
26. To ensure that samples are correctly mixed, the ratios of the summed reporter ion intensities (peptides.txt) of each individual sample within a mixture should ideally be within a range of 0.75-1.33
27. When the criteria for mixing and labelling efficiency are satisfied, thaw the samples and quench the reaction with 16 μl of 5 % hydroxylamine (solution in 50 mM HEPES) for 15 min at ∼22 °C.
28. Acidify with 10 % formic acid to ∼pH 2 (∼40 μl).
29. Combine individually the two groups of the 10 TMT labelled samples according to Figure 2. If the samples do not have the same concentration of proteins (see point 4.6), adjust proportionally the volume of each sample before mixing.
30. Dry, or partially dry to remove the organic solvent (∼30 %), by vacuum centrifugation before Sep Pak clean up. After drying for 2 h the organic solvent should have been removed. Store the dried samples at -80 °C.

**PAUSE POINT:** Dried peptides can be stored at -80 °C indefinitely.

### C18 SepPak Lite Cleanup of the Pooled Samples

**TIMING: 2 h + drying**

As in Major Step ‘C_18_ Spin Column Cleanup of Proteolytic Digests’, a C_18_ SepPak is used for each sample in conjunction with a vacuum manifold set at -2 psi, to slowly pass each solution below through the solid phase extraction device.

31. Thaw the dried, pooled TMT samples, resuspend in 0.1 % TFA and centrifuge briefly.
32. Check that the pH is ≤2, acidify with TFA, if necessary.
33. Wash the columns with 3.2 ml of acetonitrile and condition them with 1.1 ml of 50 % acetonitrile with 0.5 % acetic acid. For the following steps, make sure that the entire solution passes through the device without drying the column.
34. Equilibrate the columns with 3.2 ml 0.1 % TFA. Discard the flow through.
35. Load the samples onto the columns. Retain the flow-through.
36. Desalt with 3.2 ml of 0.1 % TFA and wash the columns with 325 μl of 0.5% acetic acid. Combine the flow through with the flow through from Step 5 and store at -80 °C in case of technical or user error during procedure and loss of sample.
37. Elute the peptides from the column by adding 1.8 ml of 50 % acetonitrile with 0.5 % acetic acid.
38. Perform a final check of the mixed sample by LC-MS/MS. In theory, 2 mg of proteins are present in each combined sample (200 μg from the 10 original samples). Dilute 10 μl of the elution in 90 μl 0.1% TFA and perform the final check by injecting 10 μl for 60 min gradient elution on the Orbitrap Fusion Lumos Tribrid mass spectrometer with an HCD MS2 fragmentation method, as described in Section 4.
39. Dry the eluted peptides by vacuum centrifugation (∼4 h, ambient). Store the dried samples at -80 °C.

**PAUSE POINT:** Dried peptides can be stored at -80 °C indefinitely.

***Alternatives:*** As in step 2, C_18_ MacroSpin column in conjunction with a centrifuge can be used to replace the C_18_ SepPak column and vacuum.

***Note:*** As in step 2, flow-through fractions of 5. and 6. are unlikely to contain peptides, they are retained in case of fault during the procedure.

***Note:*** Ideally the dried samples will remain frozen under vacuum centrifugation and have a yellow/white powder appearance. Sometimes, sample might have an oily appearance.

### Phosphopeptide Enrichment Using Sequential Enrichment of Metal Oxide Affinity Chromatography (SMOAC)

**TIMING: 1 day**

**Figure 3:**
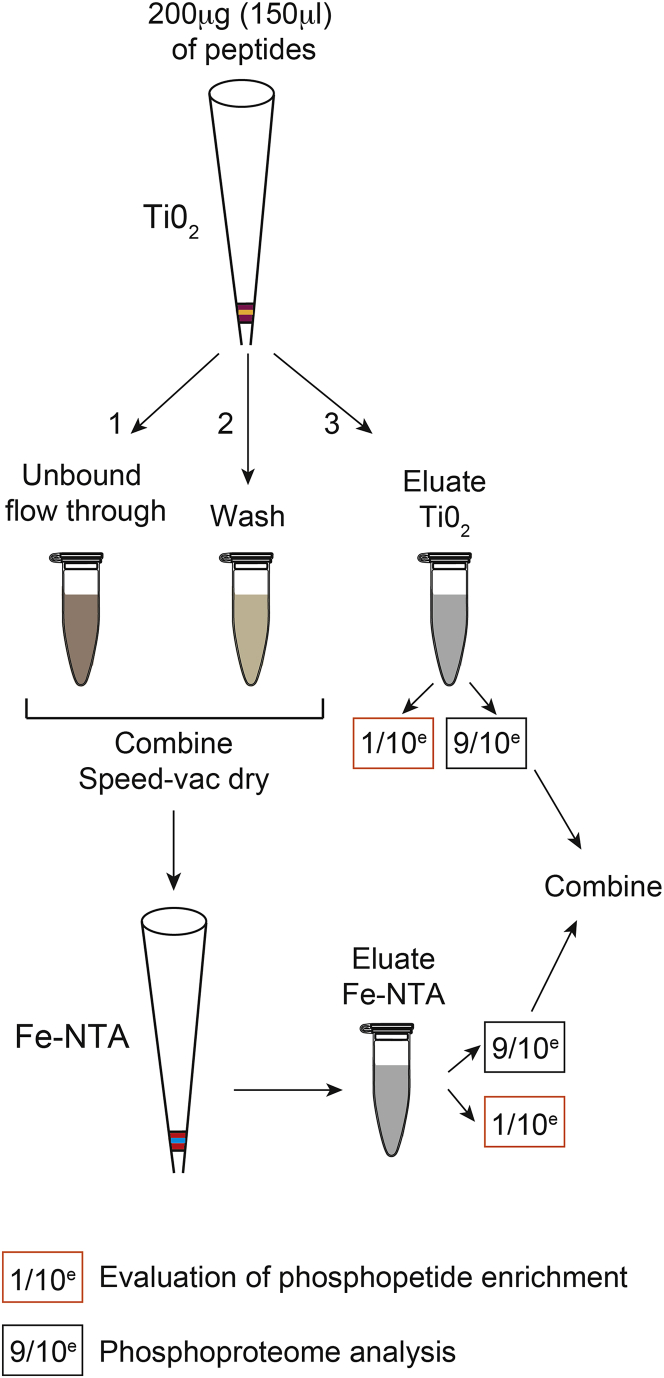
SMOAC Strategy—Combination of TiO_2_ and Fe-NTA Phosphopeptide Enrichment Kits

Sequential enrichment by Metal Oxide Affinity Chromatography (SMOAC) is used to increase the phosphopeptide enrichment. It combines titanium dioxide (Thermo Scientific High-Select™ TiO_2_ phosphopeptide enrichment kit) and ferric nitrilotriacetate (Thermo Scientific High-Select™ Fe-NTA phosphopeptide enrichment kit) phosphopeptide enrichment procedures (Figure 3).

40. Initially, enrichment is performed using the Thermo Scientific High-Select™ TiO_2_ phosphopeptide enrichment kit. Suspend the lyophilized peptide samples in 150 μl of Binding/Equilibration Buffer. Sonicate the sample tubes for 10-15 min in an ultrasonic bath until complete dissolution and centrifuge at 18000 × g for 5 min. Only soluble material should be used.
41. The manufacturer supplied protocol is followed with the exception that all unbound flow-through, wash and elution fractions are retained to be used in the next step of the protocol (Figure 3).
42. Combine the flow–through and wash fractions containing unbound peptides, this will be used during the Fe-NTA phosphopeptide enrichment step. Dry by vacuum centrifugation (∼3 h). It is normal to observe a rose-tinted jelly-like material at the end of the drying.
43. Secondary enrichment of the dried TiO_2_ flow-through fractions is completed using Thermo Scientific High-Select™ Fe-NTA phosphopeptide enrichment kit. Solubilize the dried TiO_2_ flow-through fractions in 200 μl of binding buffer using an ultrasonic bath for 15 minutes. The manufacturer supplied protocol is then followed.
44. Retain and combine the flow-through and wash fractions (which can be analyzed for protein level quantification, if required. Dry the combined flow through and eluate using vacuum centrifugation (2 h).

**CRITICAL:** Peptides should be stored as lyophilized pellets at -80 °C. Keeping them in solution at elevated pH leads to phosphate loss from phosphopeptides.

### Phosphopeptide C_18_ StageTip Concentration and Cleanup

**TIMING: 2 h**

45. Thaw the samples, re-suspended in 100 μl of 1 % TFA and sonicate for 15 min.
46. Prepare the Stage Tips (Rappsilber et al., 2007) by cutting and packing an Empore C_18_ membrane into a 200 μl pipette tip.
47. Wash the Stage Tips with 100 μl of methanol and centrifuge at 2500 rpm for 2 min. Discard the flow through.
48. Equilibrate the Stage Tips with 200 μl of 1 % TFA and centrifuge at 2500 rpm for 3 min. Discard the flow through.
49. Load the sample onto a Stage Tip and centrifuge at 2500 rpm for 3 min. Retain the flow through.
50. Wash the bound peptides with 300 μl of 1 % TFA and centrifuge at 3000 rpm for 3 min. Retain the flow through.
51. Into a fresh tube elute the peptides with 50 μl of 80 % acetonitrile and 5 % TFA at 2000 rpm for 2 min.
52. Dry the peptides by vacuum centrifugation (∼30 min).
53. Combine and store the flow-through fractions in case the peptides were not retained on the C_18_ membrane.

***Alternatives:*** C_18_ ZipTip Pipette Tips can be used to replace the use of C_18_ Stage Tips.

### Mass Spectrometry Analysis

**TIMING: 1 day**

54. Re-suspend the samples in 35 μl of 1 % TFA, sonicate for 15 min and centrifuge at 14000 rpm at 4 °C for 5 min.
55. Transfer the samples to Total Recovery glass autosampler vials.
56. Perform triplicate injections (10 μl per injection) of each mixed sample. Set the nanoLC flow rate to 250 nl/min. Separate the peptides using the following gradient: 0-5 min (2 % mobile phase B), 120 min (35 % mobile phase B), 145 min (45 % mobile phase B), 150-160 min (95 % mobile phase B), 161-180 min (2 % mobile phase B). (Phase A: 95% H2O, 5% DMSO, 0.1% Formic Acid, Phase B: 75% Acetonitrile, 5% DMSO, 20% H2O, 0.1% Formic Acid).
57. MS settings: Orbitrap mass analyser (120000 resolution), scan range 350-1500 *m*/*z*, AGC target 4.0×10^5^, maximum injection time 50 ms. Data dependent mode cycle time of 3 s between MS scans. MS2 settings: Orbitrap mass analyser 60000), activation type HCD, HCD collision energy 38 %, isolation window 0.7 *m*/*z*, first mass 100 *m*/*z*, AGC target 1.0×10^5^, maximum injection time 105 ms, include charge states 2-7, dynamic exclusion 45 s after 1 time.

## Expected Outcomes

Quantitative mass-spectrometry based proteomics can be performed using a range of quantitative techniques including, but not limited to, SILAC (stable isotope labeling by amino acids in cell culture) and LFQ (label free quantification). Due to its inherent ability to quantify ten samples simultaneously, ideal for a time course, we decided to employ isobaric labelling for quantification, specifically Tandem Mass Tags (TMT10plex) in this study. As ten samples are analyzed concurrently, this results in fewer, if any, missing values between individual time points compared to SILAC and LFQ where each time point is analyzed independently, as well as lower instrument resource requirements. We have previously published similar work using SILAC for quantification (Godfrey et al., 2017 and Touati et al., 2018) (*S. cerevisiae*), (Carpy et al., 2014 and Swaffer et al., 2016) (*S. pombe*). In budding yeast, a recent study has also taken advantage of the TMT10plex technology to study phosphoproteome variations in a time-resolved manner (Zhang et al., 2019). If all steps are followed correctly it is expected that >95 % of all peptides will be TMT labelled (this is often >99 %), and a phosphopeptide enrichment yield of >80 % will be reached (often >90 %). Optimal conditions will typically result in 5000-6000 quantified phosphosites.

## Quantification and Statistical Analysis

All data was analyzed using MaxQuant software which utilizes the embedded Andromeda database searching algorithm. MaxQuant is a freely available software (http://www.maxquant.org), is frequently updated and functions on a range of Microsoft Windows OS. A current *S. cerevisiae* proteome database can be freely downloaded from the UniProt website (http://www.uniprot.org/proteomes/). Define the two mixes as separate experiment groups in MaxQuant. Once the MaxQuant analysis has been completed, the phosphoSTY.txt output file (which contains all pertinent data for phosphosite analysis) can be imported into Perseus software for further statistical analysis and data visualization. Import the TMT reporter ion intensities as expression values into Perseus (http://www.maxquant.org/perseus/). Peptides may be phosphorylated on multiple residues. Hence, select the desired multiplicity. Remove phosphosites that were identified from the decoy and contaminant databases. Log2(x) transform all reporter ion intensity values. Normalize the data by subtracting the median value from each column (sample) to correct for mixing errors. Add categorical annotation to group samples from the two multiplexed pools, add a separate category denoting the yeast strain. Filter valid values (at least ten valid values) in at least one multiplexed pool.

Subtract the median value for each phosphorylation site separately for each multiplexed pool or use an equivalent method. For imputation, any missing values can be replaced by the mean of the two adjacent time points (t_-1_ and t_+1_ values). Data smoothing can be applied by averaging each data point with the adjacent t_-1_ and t_+1_ values. The data from the associated study have been deposited with the ProteomeXchange Consortium via the PRIDE partner repository, Project ID: PXD012860.

## Limitations

The specific protocol presented will allow the quantification of two sets of 10 samples, and thus requires imputation between TMT sets, this could be partly overcome by using 16 samples and utilizing TMT-16plex.

No primary amine containing buffers can be used prior to TMT labelling, this can be overcome by using other digestion and cleaning techniques, e.g. SP3 (Hughes et al., 2019) or FASP (Wiśniewski et al., 2009).

## Troubleshooting

### Problem

Loss of sample/low sample intensity

### Potential Solution

Return to collected flow-through fractions to identify where sample may have been lost, repeat steps ensuring correct buffers are used.

### Problem

Poor TMT labelling

### Potential Solution

If this is experienced in all samples then a primary amine-containing buffer may have been present, ensure non-primary amine-containing buffers were used, e.g. HEPES. If this is experienced in individual samples then the specific label may have become hydrolyzed, repeat labelling ensuring that buffer pH and organic:aqueous ratio is correct.

### Problem

Poor phosphopeptide enrichment/yield

### Potential Solution

Ensure that initial sample and all buffers used were at the correct pH. Ensure that the samples were desalted prior to phosphopeptide enrichment.
